# Extended-Spectrum β-Lactamase– and AmpC-Producing Enterobacteria in Healthy Broiler Chickens, Germany

**DOI:** 10.3201/eid1908.120879

**Published:** 2013-08

**Authors:** Felix Reich, Viktoria Atanassova, Günter Klein

**Affiliations:** University of Veterinary Medicine Hannover, Hannover, Germany

**Keywords:** ESBL, Enterobacteriaceae, AmpC, slaughterhouse, Proteus mirabilis, Enterobacter cloacae, Germany, bacteria, foodborne diseases, zoonoses, chickens

## Abstract

During 2010, we evaluated the presence of extended-spectrum β-lactamase– and AmpC-producing enterobacteria in broiler chickens at slaughter. Samples (70 carcasses and 51 ceca) from 4 flocks were analyzed by direct plating and after enrichment. Extended-spectrum β-lactamase producers were found in 88.6% and 72.5% of carcasses and ceca, respectively; AmpC producers were found in 52.9% and 56.9% of carcasses and ceca, respectively. Most isolates were identified as *Escherichia coli*; *Enterobacter cloacae* (cecum) and *Proteus mirabilis* (carcass) were found in 2 samples each. Molecular characterization revealed the domination of CTX-M genes; plasmidic AmpC was CIT-like. Phylogenetic grouping of *E. coli* showed types A (31.5%), B1 (20.2%), B2 (13.5%), and D (34.8%). These findings provide evidence that healthy broilers in Germany are a source for the dissemination of transmissible resistance mechanisms in enterobacteria brought from the rearing environment into the food chain during slaughtering.

Antimicrobial drug resistance is a threat for therapy failure in human medicine. The presence of enterobacteria, especially *Escherichia coli* that produces extended-spectrum β-lactamases (ESBLs), has increased during past decades in terms of the worldwide distribution of such resistance traits and of the evolution of different genes (*1*). Resistance genes of the ESBL type are mostly plasmid associated and therefore can spread among bacteria. Although chromosomal AmpC genes exist in several enterobacteria and *E. coli*, plasmid-bound types also exist that can be transferred among bacteria. These types can lead to the overall distribution of antimicrobial resistance, although the carrier bacteria are not pathogenic per se but might lead to opportunistic infections in predisposed patients because ESBL-producing *E. coli* are associated with, for example, urinary tract infection and severe systemic disease. *E. coli* infections can be nosocomial, community acquired, or foodborne. The main ESBL types are TEM, SHV, and CTX-M. Rates of CTX-M infections have increased during the last decade compared with rates of TEM and SHV infections. These enzymes confer resistance to β-lactam antibacterial drugs, particularly cephalosporins, and may be accompanied by co-resistance to drugs of other classes (*1*,*2*). Because of the ESBL resistance and associated co-resistance, empiric oral antibacterial therapy appears to be limited, especially in the community setting (*3*,*4*).

Sources of infection can be diverse. In addition to human sources of transmission in hospitals and communities, animals pose a reservoir for different pathogenic bacteria with zoonotic potential. Especially with food-producing animals, animals and humans are directly linked. Foodborne pathogens usually do not result in clinical infection of the animal host. Thus identification of sources is possible only by extensive field research in primary production and regular testing of end products. ESBL-producing enterobacteria were shown in different sources of food-producing animals at the farm and from products (*5*–*7*).

Several studies have focused on the characterization of ESBL or AmpC producers from food-production animals by testing flocks at the farm; others have focused only on fecal samples at later production steps (*8*–*11*). Less is known about the actual prevalence or diversity within single healthy broiler flocks at the slaughterhouse and the effect on meat contamination. The processing of meat contributes to overall transmission of bacteria from contamination during slaughtering and dressing, including transmission of resistant bacteria introduced at slaughter by colonized animals onto the meat product.

Our objective was to assess the prevalence of ESBL and AmpC producers in the broiler chicken–production chain in Germany in different species of the *Enterobacteriaceae* family. We focused on individual broiler flocks at the slaughterhouse level to show the introduction of enterobacteria to the slaughtering operation and transmission to the product. In addition to determining different resistance phenotypes and molecular characterization of the isolates, we evaluated the number of presumptive ESBL producers found in meat.

## Materials and Methods

### Sampling

From August through November 2010, we collected samples from housing of broiler chicken flocks at 2 different rearing sites (farms A and B) in Germany during fattening in 2 rearing periods (cycles). Each of the 4 flocks comprised up to 40.000 birds per house. To estimate flock status, we collected 2 pairs of sock swab specimens per housing 1 week before slaughter. In brief, moistened boot covers were put on the boots of specimen collectors, and specimens were collected by walking through the housing.

At the slaughterhouse, broiler carcasses and cecal samples were collected from the same flocks; the carcasses were collected after being chilled. Each flock was slaughtered at day 42 ± 1 d. For farm A, a total of 39 cecal samples and 40 carcasses were collected; for farm B, 12 cecal samples were available for cycle 1, and a total of 30 carcasses were collected for both cycles.

### Isolation

Sample preparation and isolation of ESBL- or AmpC-producing enterobacteria were conducted as follows. Sock swabs were rinsed in peptone water (Merck, Darmstadt, Germany). Cecal contents were diluted at a ratio of 1 to 10 in peptone water. Broiler carcasses were rinsed in 500 mL maximum recovery diluent (Oxoid, Wesel, Germany). Rinsates (0.1 mL) were plated onto MacConkey agar (Merck) containing 1 mg/L cefotaxime or ceftazidime (Sigma-Aldrich, Munich, Germany) and onto Brilliance ESBL Agar (Oxoid) directly; plates were incubated aerobically for 24 h at 37°C ± 0.5°C. We mixed 100 mL of carcass rinse with 100 mL of double-strength peptone water (Merck). Samples in peptone water were incubated overnight at 37°C ± 0.5°C. Enrichment cultures were streaked onto MacConkey agar and Brilliance ESBL Agar and incubated as before.

Lactose-fermenting or -nonfermenting colonies of different morphology were collected from MacConkey agar and Brilliance ESBL Agar and screened for ESBL production by disk diffusion by using cefpodoxime (10 μg), aztreonam (30 μg), ceftazidime (30 μg), cefotaxime (30 μg), and ceftriaxone (30 μg) disks (Oxoid) and confirmed by microdilution test according to Clinical Laboratory Standards Institute (CLSI) methods (*12*) by using Micronaut-S β-lactamase V test plates (Merlin Diagnostika, Bornheim-Hersel, Germany). These plates contained cefepime, cefpodoxime, ceftazidime, and cefotaxime, each with and without the addition of clavulanic acid, aztreonam, piperacillin/tazobactam, and meropenem, and meropenem/EDTA. Isolates were identified to species level by using the API 20E test kit (BioMérieux, Nürtingen, Germany).

Isolates showing cefoxitin resistance were considered presumptive AmpC producers. Production of AmpC was confirmed according to methods of Black et al. (*13*). Co-production of ESBL was confirmed by adding 200 mg/L cloxacillin (Sigma-Aldrich, Munich, Germany) to Mueller-Hinton agar (Oxoid) using disks (Oxoid) according to CLSI ESBL confirmatory tests with disks of cefotaxime and ceftazidime, with or without clavulanic acid. An increase of the inhibition zone around disks containing cephalosporine and clavulanic acid by at least 5 mm confirmed ESBL production (*14*). Other antimicrobial agents tested by microdilution (Merlin Diagnostika, Bornheim-Hersel, Germany) were ciprofloxacin, nalidixic acid, chloramphenicol, tetracycline, trimethoprim–sulfamethoxazole, gentamicin, and streptomycin.

MICs were interpreted according to the European Committee on Antimicrobial Susceptibility Testing (EUCAST) (*15*) clinical breakpoints and, for cefoxitin, nalidixic acid, and tetracycline, according to CLSI breakpoints (*12*). Epidemiologic cutoff values dividing wild-type from non–wild-type strains were evaluated according to EUCAST (*16*). The EUCAST breakpoints were chosen because of more conservative values (*17*).

### Molecular Characterization of Isolates

A subset of 76 *E. coli* isolates, confirmed ESBL or AmpC by phenotypic methods, was grouped phylogenetically by using modified triplex PCR (*18*). In brief, gene fragments of *chuA* and *yjaA* and a DNA fragment (TSPE4.C2) were amplified with primers ChuA.1 (5′-GACGAACCAACGGTCAGGAT-3′) and ChuA.2 (5′-TGCCGCCAGTACCAAAGACA-3′), fragment 279 bp; YjaA.1 (5′-TGAAGTGTCAGGAGACGCTG-3′), and YjaA.2 (5′-ATGGAGAATGCGTTCCTCAAC-3′), fragment 211 bp; and TspE4C2.1 (5′-GAGTAATGTCGGGGCATTCA-3′) and TspE4C2.2 (5′-CGCGCCAACAAAGTATTACG-3′), fragment 152 bp. PCR conditions were 3 min of initial denaturation, followed by 35 cycles at 94°C for 15 s, 60°C for 30 s, and 72°C for 45 s and final extension at 72°C for 5 min.

A representative selection of 78 isolates from cecal contents and carcasses, depending on sample type and phenotype was analyzed. The isolates were confirmed to show either an ESBL or an AmpC phenotype in the antimicrobial susceptibility tests. The presence of β-lactamase genes was confirmed by PCR with primers and conditions as reported: *bla*_TEM_ TEM-F: ATAAAATTCTTGAAGACGAAA, TEM-R: GACAGTTACCAATGCTTAATC, fragment 1,080 bp, annealing 50°C (*19*); *bla*_SHV_ SHV-F: GGGTTATTCTTATTTGTCGC, SHV-R: TTAGCGTTGCCAGTGCTC, fragment 930 bp, annealing 56°C (*20*); and *bla*_CTX-M_ CTX-M-F: SCSATGTGCAGYACCAGTAA, CTX-M-R: ACCAGAAYVAGCGGBGC fragment 585 bp, annealing 58°C (*21*). AmpC presumptive isolates were tested for the presence of AmpC gene groups by multiplex PCR according to Pérez-Pérez and Hanson (*22*): MOX-MF: GCTGCTCAAGGAGCACAGGAT, MOX-MR: CACATTGACATAGGTGTGGTGC, fragment 520 bp; CIT-MF: TGGCCAGAACTGACAGGCAAA, CIT-MR: TTTCTCCTGAACGTGGCTGGC, fragment 462 bp; DHA-MF: AACTTTCACAGGTGTGCTGGGT, DHA-MR: CCGTACGCATACTGGCTTTGC, fragment 405 bp; ACC-MF: AACAGCCTCAGCAGCCGGTTA, ACC-MR: TTCGCCGCAATCATCCCTAGC, fragment 346 bp; EBC-MF: TCGGTAAAGCCGATGTTGCGG, EBC-MR: CTTCCACTGCGGCTGCCAGTT, fragment 302 bp; FOX-MF: AACATGGGGTATCAGGGAGATG, FOX-MR: CAAAGCGCGTAACCGGATTGG, fragment 190 bp; annealing 64°C.

### Statistical Analysis

The 78 isolates were analyzed statistically to define phenotypic clusters, according to the resistance pattern. Isolates were characterized as susceptible, non–wild type, or clinically resistant according to aforementioned breakpoints for the individual antimicrobial agents and presence or absence of *bla* genes (TEM, SHV, CTX-M) and plasmidic AmpC genes. Data were analyzed by using BioNumerics software, version 5.1 (Applied Maths, Sint-Martens-Latem, Belgium) applying Pearson correlation and clustering with UPGMA (unweighted pair-group method with arithmetic mean). The cutoff for similarity was set to 80%.

## Results

All 4 broiler flocks tested positive for ESBL- or AmpC-producing enterobacteria in 2 consecutive rearing cycles. All farm-level ESBL-positive isolates were identified as *E. coli*; in AmpC-producing enterobacteria, we found *E. coli*, *Enterobacter cloacae*, or *Proteus mirabilis*. The rate of ESBL-positive isolates was higher than AmpC-positive isolates in 3 flocks. Only in flock 1 on farm B was the rate of ESBL-confirmed isolates comparatively low (1 isolate was identified as an ESBL producer); AmpC-producers dominated that cycle (data not shown). These results identify healthy broiler flocks as reservoirs of antimicrobial-resistant enterobacteria for further distribution along the food production chain in Germany.

### ESBL and AmpC Producers at Slaughter

At slaughter, ESBL- and AmpC-producing enterobacteria were found in 88.6% and 52.9% of the 70 carcasses and 72.5% and 56.9% of the 51 ceca, respectively. Most isolates were identified as *E. coli*; an AmpC-producing *P. mirabilis* isolate was detected in 1 carcass from each farm. The isolate from farm A could not be characterized further because the isolate died during storage. Furthermore, during the first cycle of farm B, *E. cloacae* strains were isolated from 2 cecal samples. Those isolates shared the same phenotype; thus only 1 isolate was characterized. At the flock level, up to 100% of the analyzed carcasses and cecal samples tested positive for ESBL-producing *E. coli* (Table 1). Overall, the presence of ESBL- or AmpC-positive enterobacteria on carcasses mirrored the presence in cecal contents.

**Table 1:** Prevalence of ESBL- or AmpC-producing enterobacteria in broiler chickens at slaughter, Germany, 2010*

Farm	Carcass				Cecum		
	No. samples	ESBL, no. (%)	AmpC, no. (%)		No. samples	ESBL, no. (%)	AmpC, no. (%)
A, 1	20	20 (100)	11 (55)		20	16 (80)	10 (50)
A, 2	20	17 (85)	9 (45)		19	19 (100)	7 (36.8)
B, 1	10	6 (60)	3 (30)		12	2 (16.7)	12 (100)
B, 2	20	19 (95)	14 (70)		0	0	0

*ESBL, extended-spectrum β-lactamase.

We tested samples of carcasses after direct plating and after enrichment. Direct plating allowed us to estimate numbers of suspected ESBL-producing *E. coli* isolates. After direct plating, 24 (38.7%) carcasses were positive. The calculated limit of detection for the direct plating was 3.7 log CFU/carcass, compared with 0.7 log CFU/carcass for the enrichment culture. The number of presumptive ESBL-producing *E. coli* isolates in samples from direct plating was 3.7–4.2 log CFU/carcass.

### Antimicrobial Resistance, β-Lactamase Genes, and Phylogenetic Typing

Testing of antimicrobial agents found 3 different resistance phenotypes. The first phenotype was ESBL only, with reduced susceptibility to at least 1 of the tested cephalosporins with sensitivity to clavulanic acid. The second was AmpC only, with cefoxitin resistance and absence of a clavulanic acid MIC reduction. The third was ESBL in the presence of AmpC production, which could not be identified directly but was shown by confirmation of the clavulanic acid effect on Mueller-Hinton agar containing 200 mg/L cloxacillin.

The resistance phenotype differed by mechanism of resistance. For all isolates, we demonstrated resistance to cefpodoxime and breakpoints above the epidemiologic cutoff value for cefotaxime and ceftazidime. Although clinical resistance to cefotaxime was more prevalent in ESBL producers, the rate of ceftazidime resistance was higher in AmpC producers and in SHV-containing isolates. Some CTX-M gene–carrying isolates only were resistant to cefepime (Table 2, Appendix; Figure, Appendix). Clinical resistance and indication of other mechanisms according to cutoff values for non–β-lactam agents differed in the isolates. Clinical resistance for tetracycline was found in >50% of isolates and to nalidixic acid and chloramphenicol in 47% and 38% of *E. coli* isolates, respectively. Because no clinical MIC for streptomycin is defined by CLSI or EUCAST (*12*,*15*), isolates were considered resistant according to epidemiologic cutoff values. Resistance was found in 76 (60%) *E. coli* isolates. Multiresistant isolates showed resistance to nalidixic acid, tetracycline, and chloramphenicol (streptomycin) in most cases.

**Table 2:** MIC_50_ and MIC_90_distribution in *Escherichia coli* of ESBL or AmpC producers in broiler chickens, Germany, 2010*

	FEP	FEP/CLA	CTX	CTX/CLA	FOX	CPD	CPD/CLA	CAZ	CAZ/CLA	ATM	TZP	CIP	NAL	CHL	TET	TMP/SXT	MEM	GEN	STR
Breakpoint, µg/mL	>8	–	>4	>4	>32†	>2	–	>8	–	>8	>32	>2	>32	>16	>16†	>8	>16	>8	–
ECOFF	<0.125	–	<0.25	<0.25	<8	<2	–	<0.5	–	<0.25	<8	<0.064	<16	<16	<8	<1	<0.125	<2	<16
Total, n = 76																			
MIC_50_	2	<0.25	16	1	8	>32	1	16	0.5	2	2	<0.06	8	8	16	0.25	<0.063	0.5	32
MIC_90_	32	1	>32	16	>64	>32	>32	>32	32	8	4	1	>128	64	>16	>8	<0.063	1	>64
ESBL, n = 42																			
MIC_50_	8	<0.25	32	<0.25	4	>32	<0.25	8	<0.25	2	<1	0.125	16	8	>16	0.25	<0.063	0.5	32
MIC_90_	>32	<0.25	>32	1	8	>32	1	32	0.5	4	2	2	>128	64	>16	>8	<0.063	1	>64
ESBL, AmpC, n = 13																			
MIC_50_	2	<0.25	16	16	64	>32	>32	32	16	4	4	0.25	128	32	>16	0.125	<0.063	0.5	>64
MIC_90_	8	0.5	32	32	>64	>32	>32	>32	32	8	4	0.5	128	64	>16	>8	<0.063	1	>64
AmpC, n = 21																			
MIC_50_	0.5	0.5	16	16	64	>32	>32	32	16	2	4	<0.063	2	4	1	<0.063	<0.063	0.5	4
MIC_90_	1	1	32	16	>64	>32	>32	32	32	>8	8	0.25	128	16	16	0.25	<0.063	1	>64

*ESBL, extended spectrum β-lactamase; FEP, cefepime; FEP/CLA, cefepime/clavulanic acid; CTX, cefotaxime; CTX/CLA, cefotaxime/clavulanic acid; FOX, cefoxitin; CPD, cefpodoxime; CPD/CLA, cefpodoxime/clavulanic acid; CAZ, ceftazidime; CAZ/CLA, ceftazidime/clavulanic acid; ATM, aztreonam; TZP, piperacilin/tazobactam; CIP, ciprofloxacin; NAL, nalidixic acid; CHL, chloramphenicol; TET, tetracycline; TMP/SXT, trimethoprim/sulfamethoxazole; MEM, meropenem; GEN, gentamicin; STR, streptomycin; ECOFF, epidemiologic cutoff value.

**Figure:**
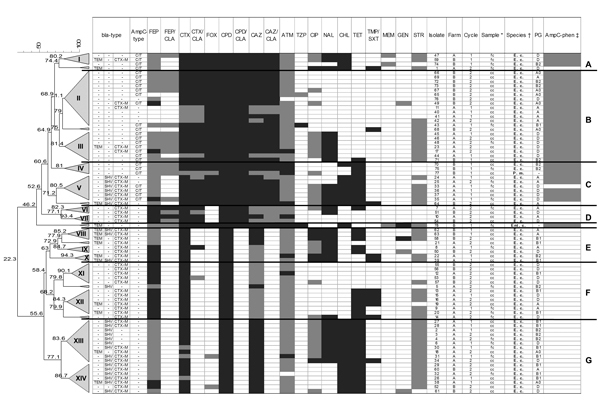
Phenotype distribution and dendrogram of 78 enterobacteria isolates from broiler chickens at the slaughterhouse, Germany, 2010. The dendrogram was generated by the unweighted pair-group method with arithmetic mean and Pearson correlation; trees were collapsed at a cutoff value of 80%. CEP, cefepime; FEP/CLA, cefepime/clavulanic acid; CTX, cefotaxime; COX, cefoxitin; CPP, cefpodoxime; CTX/CLA, cefpodoxime/clavulanic acid; CAZ, ceftazidime; CAZ/CLA, ceftazidime/clavulanic acid; ATM, aztreonam; TZP, piperacilin/tazobactam; CIP, ciprofloxacin; NAL, nalidixic acid; CHL, chloramphenico; TET, tetracycline; TMP/SXT, trimethoprim/sulfamethoxazole; MER, meropenem; GEN, gentamicin; STR, streptomycin; TEM, CTX-M, and SHV are extended spectrum β-lactamase (ESBL) types. Light gray, non–wild-type according to ESBL; ECOFF, epidemiologic cutoff value (*16*); dark gray, clinically resistant (*12**,**15*); *fc, cecal content; cc, carcass after chilling; †E.c., *Escherichia coli*; P.m., *Proteus mirabilis*; En.c., *Enterobacter cloacae*; ‡AmpC-test (*13*); gray, positive test; ESBL confirmation test with cloxacillin containing agar; A–G: clades; I–XIV, clusters.

We analyzed *bla* genes by PCR. We found all 3 *bla* genes (*bla*TEM, *bla*SHV, and *bla*CTX-M), with *bla*CTX-M being the most prevalent. 

A total of 78 (95%) isolates tested by PCR were positive for at least 1 resistance gene. A single resistance gene was detected in 42 (54%) isolates; 32 (41%) isolates showed 2 or 3 resistance genes.

We identified ESBL producers that carried an additional plasmidic AmpC in 9 (12%) isolates from different samples. The *bla*CTX-M was identified in 55 (89%) typed ESBL producers; *bla*TEM and *bla*SHV were found in 27% and 47%, respectively. In AmpC producers, only CIT-like genes were found in 36 (75%) cefoxitin-resistant or AmpC-positive isolates. The CIT group of AmpC β-lactamases includes CMY with CMY-2 as the most prevalent member (*23*,*24*). Eight isolates of *E. coli* indicating an AmpC phenotype tested negative for plasmidic AmpC. 

Phylogenetic typing revealed the 4 groups of *E. coli*. The types found most often were D and A, followed by B1 and, to a lesser extent, B2 (Table 3). The distribution was similar in carcasses of both flocks. In ceca, this distribution also was reflected for farm A; the limited number of isolates from farm B provided evidence only of B2 and D.

**Table 3:** Phylogenetic group distribution of extended spectrum β-lactamases– and AmpC-producing *Escherichia coli* in broiler chickens, Germany, 2010

Phylogenetic group	At farm, no. (%)		At slaughter, no. (%)					Total, no. (%)
			Ceca			Carcass		
	Farm A	Farm B	Farm A	Farm B		Farm A	Farm B	
A	4 (44)	1 (25)	4 (22)	0		9 (30)	10 (40)	28 (32)
B1	2 (22)	2 (50)	5 (28)	0		8 (27)	1 (4)	18 (20)
B2	1 (11)	0	2 (11)	2 (67)		3 (10)	4 (16)	12 (14)
D	2 (22)	1 (25)	7 (39)	1 (33)		10 (33)	10 (40)	31 (35)

Comparison of isolates showed 2 major groups with 7 clades (A–G) and 14 clusters at a cutoff at 80% similarity (I–XIV) and 7 isolates that did not cluster with other strains (Figure, Appendix). The major groups were identified by the phenotype of resistance or susceptibility to clavulanic acid. The clades included strains of different origin because isolates from both farms and/or different sample types clustered.

The 2 other enterobacteria found clustered in separate branches. *P. mirabilis* isolates clustered with *E. coli* isolates in cluster IV, which might be due to the resistance inferred by the same resistance gene of CIT type possibly resulting from interspecies plasmid transfer. The single *E. cloacae* strain did not cluster with other isolates but clustered in the group with clavulanic acid–resistant isolates. This strain was positive in the AmpC test. Absence of plasmidic AmpC indicates inducible enzymes. In addition, the strain contained TEM β-lactamase.

## Discussion

Bacteria in food-producing animals are spread through the food chain, which is important in terms of food shelf life and for transmission of pathogenic bacteria to the consumer. High numbers of bacteria can reduce shelf life and increase early food spoilage. Foodborne pathogens and bacteria with zoonotic potential are in focus worldwide because of immense health loss and costs that arise from foodborne infection associated with bacteria, such as *Salmonella* sp. and *Campylobacter* spp. (*25*).

Antimicrobial resistance is also of concern because of the limitation and even the risk for loss of effective antimicrobial treatment of infections; evidence exists that resistance from enterobacteria, such as *Salmonella* sp. and *E. coli* of animal origin can be transferred to humans (*26*,*27*). Food-producing animals may play a role in this process, and the food production chain needs to be evaluated to identify the potential for transmission pathways. Animals infected with antimicrobial-resistant strains of bacteria seem to be linked directly with human bacterial strains of *E. coli* (*28*–*30*).

Studies that focus on ESBL-producing *E. coli* at slaughter have found prevalences of 42.1% in feces of broilers and of 60% in chicken carcasses at the retail level in Portugal, but these studies have not included a flock attribution analysis (*9*,*31*). Results from different studies are sometimes difficult to compare because of different isolation and testing methods.

Total *E. coli* or coliforms usually are found in 100% of broiler carcasses at slaughter, at concentrations of 2.5 log CFU/mL for *E. coli* and 2.8 log CFU/mL for coliforms in rinse (100 mL/carcass), resulting in ≈4–5 log CFU/carcass (*32*). The percentage of samples positive for ESBL enterobacteria after direct plating and their numbers in this study were lower than those in reports of total *E. coli*, but in the view of possible genetic transfer between bacteria, even low numbers of bacteria harboring mechanisms of antibacterial drug resistance are relevant.

Moreno et al. (*11*) compared the proportion of *E. coli* with reduced susceptibility to expanded-spectrum cephalosporins with the total *E. coli* in the feces of healthy food animals. The resistant population in broilers was 4.3%. Horton et al. (*33*) estimated shedding densities of presumptive CTX-M *E. coli* for cattle, pigs, and chicken in the United Kingdom, where the latter showed higher shedding rates than did the red meat species. Although those studies described the numbers or proportions of resistant *E. coli* in feces of healthy animals, our study focused on total enterobacteria expressing the ESBL phenotype on carcasses at the end of slaughtering.

Fecal contamination, a known problem during broiler production, can lead to contamination of the meat with foodborne pathogens, such as *Salmonella* sp. and *Campylobacter* spp. (*34*,*35*). The role of shedding of bacteria through feces, which leads to contamination of carcasses during slaughtering, was evident in our study. Consequently, a high prevalence of ESBL-producing bacteria in colonized flocks could be shown. As a result, a considerable proportion of broilers were surface contaminated with ESBL-producing bacteria during slaughter.

In connection with the farm isolates, it is evident that strains from the 4 phylogenetic groups were already present during rearing of the poultry. A similar distribution also was found in other countries, where type A or type D dominated in poultry, and type B2 was present at a lower rate (*29*,*36*). Especially groups B2 and, to a lesser extent, type D are of public health concern. Those are supposed to contain strains of higher pathogenic character resulting from more virulence traits. B2 strains can be found in diseased and in healthy poultry and could have zoonotic potential through direct bird-to-human transmission or as genetic reservoir (*37*,*38*). This fact is even more important when increased virulence is paired with antibacterial drug resistance.

The sampled farms were geographically separate, and their poultry were supplied by different companies; however the farms were situated in a region with a high density of food animal production, which could indicate a common source, or selective pressure. At the cluster level, the picture was more diverse, and several clusters contained isolates unique to the individual farms. Isolates from the different sample types (cecal contents, carcasses) clustered, which indicates fecal contamination during the slaughtering process.

Co-resistance to non–β-lactam antibacterial drugs was most often associated with ESBL genes and was less prevalent in AmpC isolates (Table 2, Appendix; Figure, Appendix). Resistance to nalidixic acid found in several clusters (I, III, V, VIII–IX, and XIII), was usually combined with reduced susceptibility to ciprofloxacin. This resistance was present in isolates of various ESBL or AmpC gene combinations. High levels of nalidixic acid–resistant isolates are considered a first step in mutation to fluorquinolone-resistant strains (*8*). Reduced susceptibility to ciprofloxacin in the tested isolates, together with nalidixic acid resistance, indicates this process. Resistance to chloramphenicol and tetracycline, on the other hand, was most often linked with isolates carrying *bla*_SHV_ alone or in combination with resistance to nalidixic acid. The co-resistance and the rate of reduced susceptibility are comparable to findings in a recent study by Dierikx et al. (*5*) and may be caused by similar treatment regimens in conventionally reared broilers in Germany and the Netherlands. Phylogenetic groups were not linked to definite resistance patterns, but isolates of the clinically important B2 group were found with either SHV- or CIT-like genes. Overall, the clustering showed a diversity of resistance phenotypes and *bla* gene combinations. These were present in different *E. coli* isolates according to the phylogenetic typing.

The ESBL gene families identified showed distributions comparable with distributions recently reported in Europe and other continents. TEM-52, SHV-12, and CTX-M-1 are the most often reported types from the food animal reservoir (*2*,*39*). CIT-like was the only AmpC type found. The absence of AmpC genes in some phenotype-confirmed isolates might indicate a different mechanism of resistance, probably attributable to overexpression of chromosomal AmpC, which usually results from mutations in the promoter/attenuator region (*40*).

Cephalosporin-resistant enterobacteria isolates were prevalent in the broiler flocks studied. Furthermore, colonization of broilers during rearing correlated with considerable contamination of broiler meat at the slaughterhouse. Isolates in the animals’ feces are distributed to the carcasses during the slaughtering operation by fecal contamination. This is a vital point when assessing the transmission potential through the food chain. Therefore, broilers seem to be an important reservoir for enterobacteria with transmissible mechanisms of resistance. In addition to ESBL-producing strains, a considerable number of isolates contained plasmidic AmpC of CIT type.

Phylogenetic characterization of *E. coli* isolates identified possible extraintestinal pathogenic group B2 strains with low prevalence. Their presence, together with the various resistance phenotype, should be observed further to evaluate the distribution and effect on public health.
